# Induction of Microglia Activation after Infection with the Non-Neurotropic A/CA/04/2009 H1N1 Influenza Virus

**DOI:** 10.1371/journal.pone.0124047

**Published:** 2015-04-10

**Authors:** Shankar Sadasivan, Mark Zanin, Kevin O’Brien, Stacey Schultz-Cherry, Richard J. Smeyne

**Affiliations:** 1 Department of Developmental Neurobiology, Saint Jude Children’s Research Hospital, Memphis, Tennessee, 38105, United States of America; 2 Department of Infectious Diseases, Saint Jude Children’s Research Hospital, Memphis, Tennessee, 38105, United States of America; Emory University, UNITED STATES

## Abstract

Although influenza is primarily a respiratory disease, it has been shown, in some cases, to induce encephalitis, including people acutely infected with the pandemic A/California/04/2009 (CA/09) H1N1 virus. Based on previous studies showing that the highly pathogenic avian influenza (HPAI) A/Vietnam/1203/2004 H5N1 virus was neurotropic, induced CNS inflammation and a transient parkinsonism, we examined the neurotropic and inflammatory potential of the CA/09 H1N1 virus in mice. Following intranasal inoculation, we found no evidence for CA/09 H1N1 virus neurotropism in the enteric, peripheral or central nervous systems. We did, however, observe a robust increase in microglial activity in the brain characterized by an increase in the number of activated Iba-1-positive microglia in the substantia nigra (SN) and the hippocampus, despite the absence of virus in the brain. qPCR analysis in SN tissue showed that the induction of microgliosis was preceded by reduced gene expression of the neurotrophic factors *bdnf*, and *gdnf* and increases in the immune modulatory chemokine chemokine (C-C motif) ligand 4 (*ccl4*). We also noted changes in the expression of transforming growth factor-1 (*tgfβ1*) in the SN starting at 7 days post-infection (dpi) that was sustained through 21 dpi, coupled with increases in *arginase-1* (*arg1*) and *csf*1, M2 markers for microglia. Given that neuroinflammation contributes to generation and progression of a number of neurodegenerative disorders, these findings have significant implications as they highlight the possibility that influenza and perhaps other non-neurotropic viruses can initiate inflammatory signals via microglia activation in the brain and contribute to, but not necessarily be the primary cause of, neurodegenerative disorders.

## Introduction

Influenza A viruses infect a number of different animals, ranging from birds and pigs to humans [1]. In addition to the well-documented respiratory effects, acute infection in humans can lead to the development of encephalitis [2,3] with neurological symptoms ranging from headaches to coma to death [4]. These neurological symptoms have manifested following a number of H1N1 infections, including the 1918 Spanish flu and 2009 pandemic [2,5,6]. Although the neuropathological aspects of encephalitis following acute infection have been well described, the mechanism of its induction is controversial, with some attributing it to a primary response in reaction to the physical presence of the virus within the CNS, while others suggesting it is a result of an adaptive immune response, secondary to systemic infection [7,8].

Previous studies in our lab demonstrated that the highly pathogenic avian influenza (HPAI) VN/1203 H5N1 influenza virus was neurotropic (enters the CNS) and induced a significant inflammatory response throughout the brain. This encephalitic response was characterized by an increase in the number of morphologically activated microglia and inflammatory cytokines and chemokines [9,10]. Within the brain, microglia function by constantly surveying the CNS and when they sense altered homeostasis, such as the presence of an inflammagen, they undergo both a physical change (a process known as activation) and a secretory program that is thought to be specific to the type of insult [11]. In general, microglial induction is thought to be protective, with the goal of eliminating the encountered pathological species. However, this activation can also have unintended consequences, leading to neurotoxicity [12]. For this reason, mechanisms exist to deactivate the microglial response. Transforming growth factor-beta 1 (TGFβ1) is one molecule with anti-inflammatory properties that has been suggested to function in this role [13]. TGFβ1signaling is mediated by transmembrane serine/threonine protein kinases and consists of the ligand binding to the TGFβ1receptor present on the surface of the microglia that results in phosphorylation and translocation of Smad2 and Smad3 to the nucleus to regulate multiple signaling pathways [14]. Additionally, TGFβ suppresses class II MHC on microglia, which leads to a suppression of cytokine release [15].

In the current study, we assessed the neurological and microglia changes that occurred in the CNS of C57BL/6J mice following intranasal infection with the pandemic CA/09 H1N1). We examined two regions of the brain, the substantia nigra (SN) and the hippocampus, which are commonly affected in cases of Parkinson’s disease and Alzheimer’s disease, respectively. Though, CA/09 H1N1 influenza virus was found to be non-neurotropic, we did observe a robust induction of microglial activity and alteration in gene expression of both neurotrophic and immune-related genes. This work demonstrates that non-neurotropic influenza viruses have the ability to induce microglia activation that can potentially induce both short- and long-term neuropathological consequences [16,17].

## Results

### CA/09 H1N1 influenza virus infection leads to microglia activation in the substantia nigra pars *compacta* (SNpc) and the hippocampal dentate gyrus

**Fig 1:**
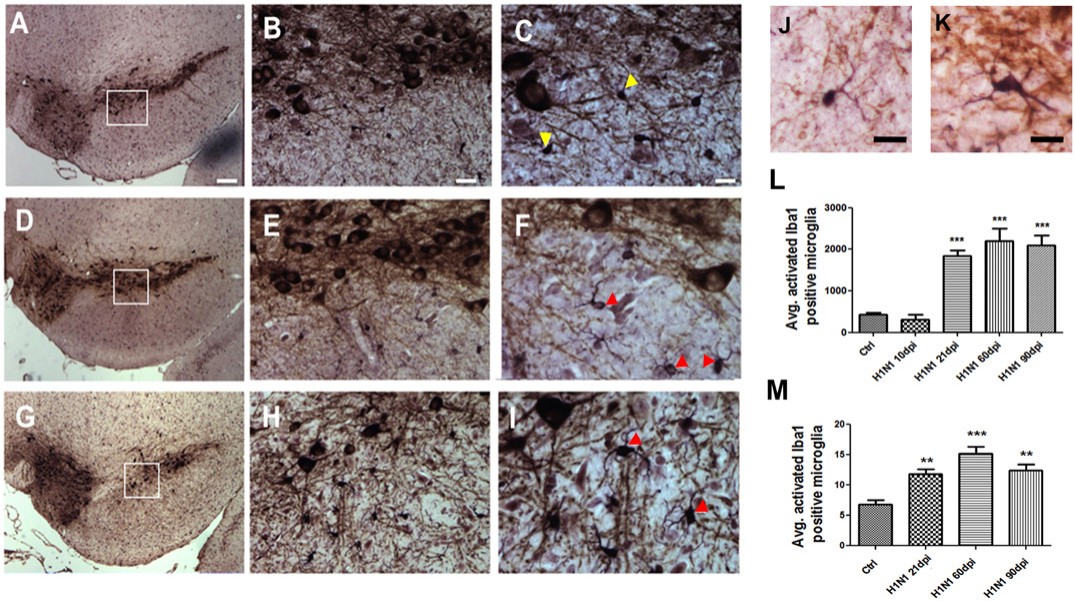
H1N1 infection results in increased numbers of activated Iba-1 positive microglia in the substantia nigra *pars compacta* (SNpc) region of the brain. Representative images presented here of the SNpc from saline-treated (A-C) or H1N1 [21dpi (D-F) & 60 dpi (G-I)-infected] demonstrate Iba-1 positive microglia with different morphology (yellow arrowheads-resting; red arrowheads-activated). The inset box demonstrates the magnified regions of the SNpc (20X and 40X, respectively) to better demonstrate the morphology of Iba-1 positive microglia. Magnified images of Iba-1 positive microglia show resting (J) and activated morphology (K). Stereological estimates of Iba-1 positive activated microglia in the SNpc (L) and dentate gyrus of the hippocampus (M) are graphically represented. Graph demonstrates stereologically acquired numbers of activated morphology Iba-1 positive microglia in the SNpc following 21 dpi, 60 dpi and 90 dpi compared to saline administered controls (n = 5). All statistics were one-way ANOVA followed by Dunnett’s post-hoc comparisons. ***p≤0.001 compared to control, **** p≤0.0001 compared to control. Scale Bars: A,D,G 100 μm, B,E,F 20 μm, C,F,I, 10 μm, J,K 10 μm.

Lightly anesthetized C57BL/6J mice were intranasally inoculated with 10^3^ TCID_50_ non-mouse adapted CA/09 H1N1 (CA/09) virus. Examination of the CNS from 7 to 90 days post-infection (dpi) demonstrated a diffuse neuroinflammatory encephalopathy characterized by a qualitative increase in the number of activated Iba-1+ microglia in the SNpc (Fig 1A–1I)and hippocampus (S2 Fig). Based on this qualitative observation, we performed a stereological assessment of the number of Iba-1 positive resting (Fig 1J) and activated microglia (Fig 1K) in both the midbrain SNpc and the hippocampal dentate gyrus, two regions of the brain that were shown to be an HPAI H5N1 influenza virus infection [18,19]. Starting at 21 dpi and persisting through 90 dpi there was a significant increase in the number of morphologically-activated Iba-1-positive microglia in mice intranasally infected with CA/09 virus as compared to mice intranasally administered saline (controls) in both SNpc (Fig 1L) and hippocampal dentate gyrus (Fig 1M). Stereological assessment of the dopamine neurons (TH- and Nissl- positive) in the SN did not yield any significant loss at 10, 21, 60 or 90 dpi compared to saline controls (S1 Fig)

### CA/09 H1N1 influenza virus is not neurotropic

**Fig 2:**
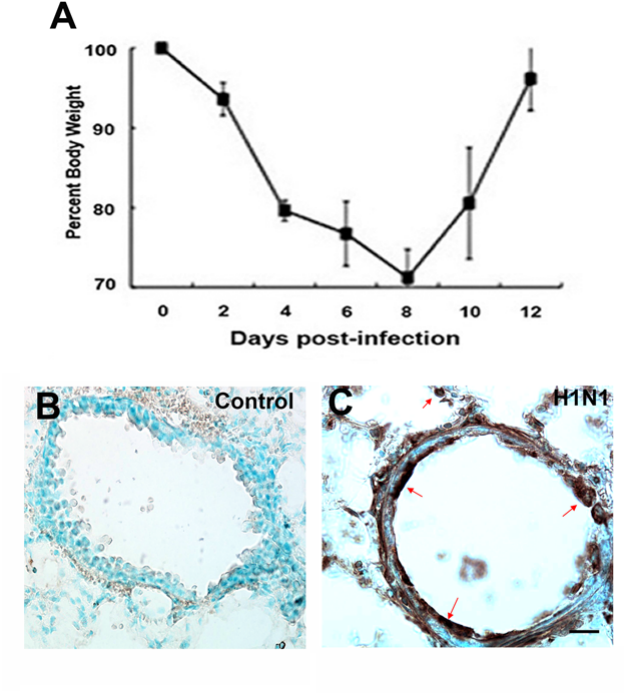
Positive CA/09 H1N1 infection results in weight loss in infected mice and is detected using anti-NP. A) 8-weeks old female C57BL/6J mice infected intranasally with CA/09 H1N1 exhibited a 30% loss of body weight; considered to be a positive sign of infection (n = 20). (B,C) Representative images of lung sections immunostained with influenza nucleoprotein antibody (NP) from saline administered controls and CA/09 infected (3 dpi) mice, respectively. Positive presence of viral infection was detected using anti-NP in lungs at 3 dpi (arrows). Scale Bars B,C = 25 μm.

CA/09 virus causes significant morbidity in C57BL/6J mice, as evidenced by the loss of approximately 30% body weight (Fig 2A) by 8 dpi. Lung infection was confirmed by immunostaining for the highly conserved viral nucleoprotein (NP) at 1, 3 and 7 dpi (Fig 2B and 2C) and levels quantitated by TCID_50_ analysis demonstrating that titers reached 10^4.3^ at 3 dpi elevating slightly to 10^5.3^ by 7 dpi. (Table 1). Infection of mice with the same dose among different cohorts yielded similar results with an average mortality of 15% per infection. Though weight loss and other physical attributes such as hunched posture and disheveled coat appearance were observed following infection, mice did not display any overt neurological symptoms during peak infection To determine if the CA/09 virus was neurotropic, we examined the enteric, peripheral and central nervous system (CNS) at 1, 3, 7, 10 and 21 dpi for the presence of influenza nucleoprotein (NP). Unlike neurotropism seen following HPAI H5N1 infection [18], we did not observe NP-immunopositive viral particles in any division of the nervous system at the times examined. The lack of intraparenchymal virus in the CNS was also confirmed using quantitative reverse transcription polymerase chain reaction (qPCR) to measure expression of the influenza M1 viral RNA [19,20] in different brain regions (cortex, substantia nigra, striatum and hippocampus) at 7 and 21dpi. The absence of detectable M1 mRNA supported our negative immunohistochemical findings (Table 2). These observations are in line with other studies demonstrating that the CA/09 virus is non-neurotropic [21].

**Table 1:** Lung viral titers following infection.

Strain	Days post-infection	TCID_50_ dose
C57BL/6J	0 dpi	1x10^3^
C57BL/6J	3 dpi	10^4.75^, 10^4.5^, 10^3.75^
C57BL/6J	7 dpi	10^5.5^, 10^5.5^, 10^5^

Influenza titers were increased in the lungs post-infection. Influenza titers were obtained in lungs infected with CA/09 at 0, 3 and 7 dpi (n = 3). The titers are presented as TCID_50_.

**Table 2:** Influenza M1 mRNA expression in different regions of the brain.

	SN (cycles)	Hippocampus (cycles)	Striatum (cycles)	Cortex (cycles)	CA/09 H1N1 positive-ctrl (cycles)
Ctrl day 1	ND	ND	ND	ND	**20.37**
Ctrl day7	ND	ND	ND	ND	ND
Ctrl day21	ND	ND	ND	ND	ND
H1N1 day 7	ND	ND	ND	ND	ND
H1N1 day 21	ND	ND	ND	ND	ND

ND Not detected. Influenza gene M1 mRNA expression in different regions of the brain. Influenza matrix (M1) protein mRNA expression was determined using qPCR in the substantia nigra (SN), hippocampus, striatum and cortex regions of the brain following CA/09 H1N1 infection at 7 and 21 dpi (n = 3). A diluted solution of the CA/09 H1N1 was used as a positive control.

### Blood brain barrier (BBB) is not compromised and T-cell infiltration into the brain parenchyma is absent following CA/09 H1N1 infection

**Fig 3:**
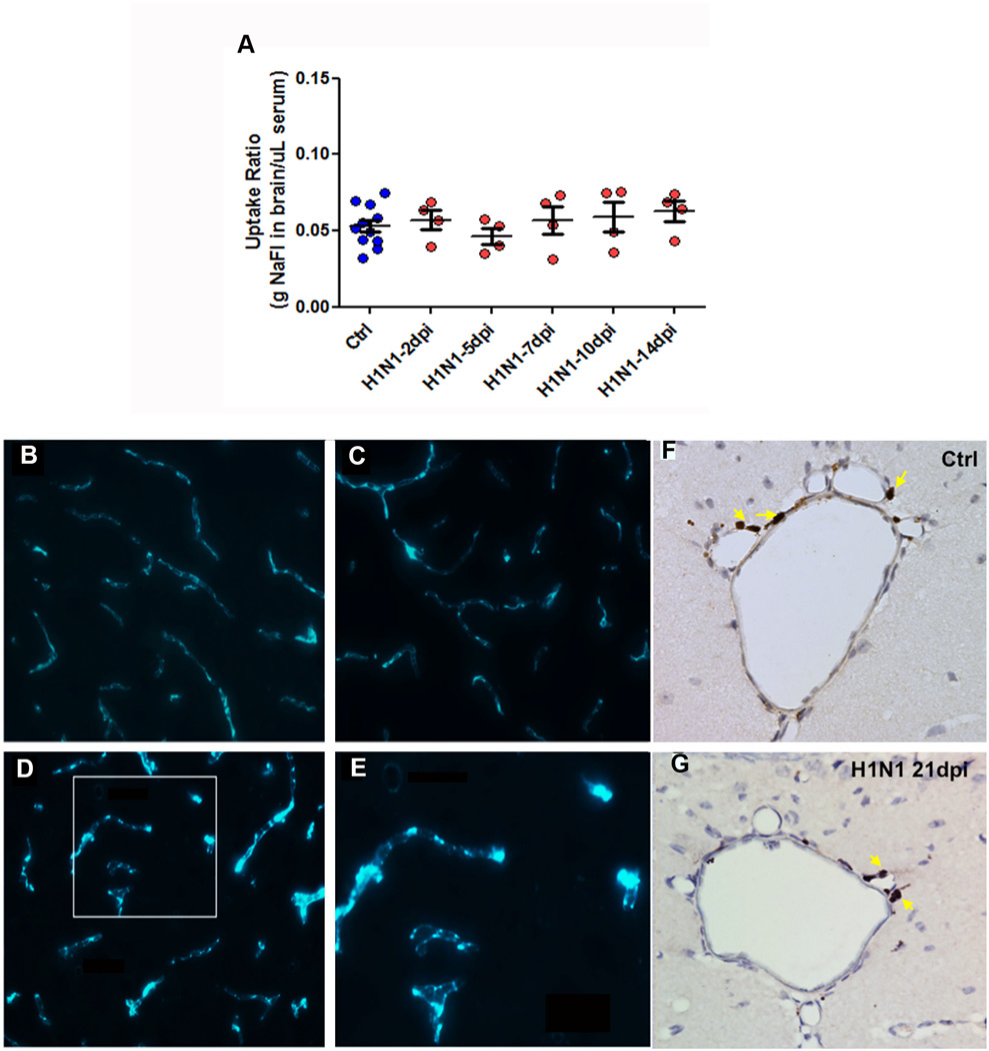
Blood brain barrier (BBB) is uncompromised accompanied with lack of T-cell infiltration in the brain parenchyma following CA/09 H1N1 infection. (A) Blood brain barrier integrity was investigated by plotting the sodium fluorescein dye uptake ratio in the brain/μl serum in mice either infected with CA/09 (H1N1) or administered saline (Ctrl) at 2, 5, 7, 10 and 14 dpi (n = 4/infection group). One-way ANOVA statistical test did not yield any significant differences between infected and saline groups. Representative images (n = 3) demonstrating FITC-conjugated albumin contained in the blood vessels of brain parenchyma among animals administered saline (B) and H1N1-infected (C). Lipopolysaccharide (LPS; 2x3mg/kg) was used as a positive control to cause BBB permeability (D). Arrow indicates permeation of the FITC-conjugated album into the parenchyma. The inset box has been magnified to better represent the presence of fluorescein signal in the parenchyma (E). Also, immunostaining with anti-CD3 revealed the concentration of T-cells along the blood vessels or around the choroid plexus regions of the brain rather than the brain parenchyma in both saline-controls (F) and H1N1-infected (G). Arrows indicate anti-CD3 positive T-cell population around blood vessels. Representative images were acquired at 40X magnification (n = 3). Scale bars: A,B,C = 50 μm, B,D = 25μm, E,F = 100μm.

Given the increased number of activated microglia in the CNS of CA/09 virus infected mice, we hypothesized that systemic inflammatory signaling increased during infection might be responsible for the morphological transformation of microglia. One way for peripheral cytokines/chemokines to interact with the CNS is through a leaky blood brain barrier (BBB). To examine the integrity of the BBB, we injected 10% (w/v) sodium fluorescein intraperitoneally in mice to determine if there was an increased fluorescein uptake in brain tissue due to leaky blood vessels [22]. The absence of increased fluorescein intensity uptake ratio in the brain normalized against serum following CA/09 infection from 2, 5, 7, 10 and 14 dpi, suggested that the infection did not disrupt the BBB (Fig 3A). Another method used to investigate BBB compromise was through the intravenous administration of albumin-FITC in mice and qualitatively assess disruption of the blood brain barrier by observing for FITC luminescence in the brain parenchyma. In mice administered H1N1, the FITC was visualized within the lumen of the blood vessels without any leakiness into the surrounding parenchyma similar to saline administered controls (Fig 3B and 3C). Brains obtained and sectioned 24 hours following administration of lipopolysaccharide (LPS; 2x3 mg/kg), an endotoxin known to disrupt the BBB [23], were used as positive controls to observe for FITC leakage into brain tissue (Fig 3D and 3E).

One other potential cell type that could act as a signal intermediary is the T-cell. T-cells, known to be increased in the periphery during infection and secrete a variety of factors some of which activate microglia [24], can penetrate into the CNS by a variety of processes [25]. To empirically examine brains for the presence of T-cells, we processed brains from CA/09 virus infected mice to detect the presence of CD3-positive cells, a pan-T-cell marker. We found no qualitative difference in T-cell numbers or location in H1N1-infected mice at 7, 10, 14 and 21 dpi as compared to saline administered controls. In each case, we observed CD-3+ T-cells along the ventricular walls, without overt presence within the brain parenchyma (Fig 3F and 3G).

### Altered expression of neurotrophic factors and chemokines in the CNS during CA/09 virus infection

**Fig 4:**
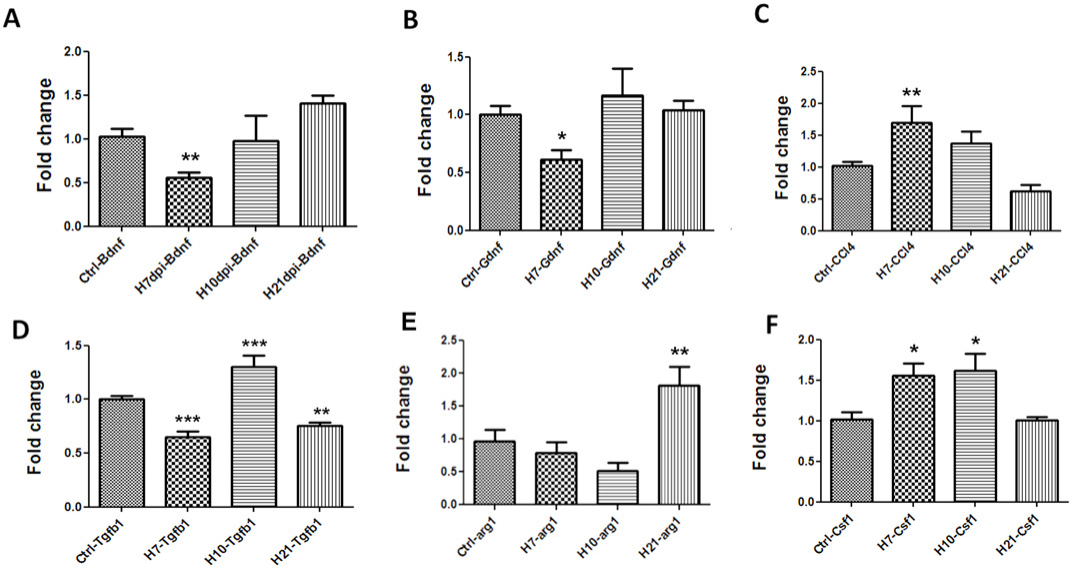
CA/09 H1N1 infection causes gene expression changes in the substantia nigra. Fold change values in mRNA expression presented are normalized against saline controls (Ctrl) in the substantia nigra at 7 (H7), 10 (H10) and 21 (H21) days post-infection (dpi). The genes probed and represented here include A) *bdnf*, B) gdnf, C) *ccl4*, D) *tgfβ1*, E) *arg1* and F) *csf1*. One-way ANOVA statistical analyses yielded significant differences between controls and infected groups (n = 5). *** indicates p≤0.0001 compared to controls; ** indicates p≤0.001 compared to controls; * indicates p≤0.01 compared to controls.

**Fig 5:**
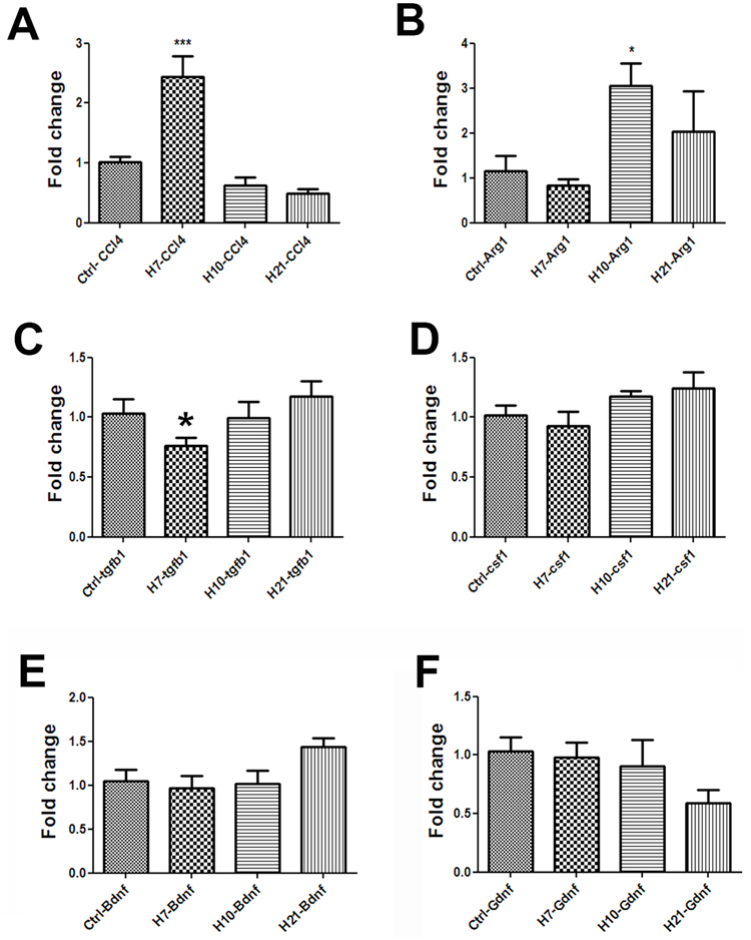
Gene expression changes in the hippocampus at 7 days post CA/09 H1N1 infection. Fold change in mRNA expression of A) *ccl4*, B) *tgfβ1*, C) *arg1*, D) *csf1*, E) *bdnf* and F) *gdnf* in the hippocampus 7, 10 and 21 days post-H1N1 infection normalized against saline controls (Ctrl). Data is presented as means ± S.E.M. * indicates p≤0.01 compared to control (n = 5). β-actin was used as a housekeeping control gene.

Given the increased number of activated microglia in the CNS in the absence of viral neurotropism or increased peripheral T-cell presence in the CNS, we examined mRNA expression of a number of genes documented to affect microglial activity. Seven days after intranasal CA/09 inoculation we observed, a down-regulation of brain-derived neurotrophic factor (*bdnf)* (Fig 4A) and glia-derived neurotrophic factor (*gdnf)* in the SN, (Fig 4B), whilst expression of the immune modulatory factor chemokine ligand 4 (*ccl4)* gene was increased (Fig 4C). These alterations in gene expression were statistically non-significant by 10 dpi. Examination of other genes known to modulate microglial activity, including the toll-like receptors (*tlr1*, *tlr3*, *tlr4*, *tlr6 and tlr9)*, *gfap*, *il-1b*, *il-6*, *ptgs2 and tnf-α* did not show any statistically significant alterations in expression levels following CA/09 virus infection at 7, 10 or 21 dpi in the SN (S1 Table). In the hippocampus, the expression of both *bdnf* (Fig 5E) and *gdnf* (Fig 5F) genes were unaltered, although the expression of *ccl4* was significantly increased at 7 dpi, however the gene expression levels reached statistically non-significant levels by 10 dpi (Fig 5A).

### *Tgfβ1* signaling is altered following CA/09 infection in the SN

Given that we did not observe alterations in the expression of a number of pro-inflammatory genes, we investigated expression of *tgfβ1*, a gene that has been implicated in attenuating the inflammatory response in the CNS; especially via “deactivation” of microglia [26,27]. We examined mRNA levels of *tgfβ1* in both the SN and the hippocampus at 7, 10 and 21 dpi. In SN, compared to saline control mice, *tgfβ1* expression was significantly down-regulated at 7, increased at 10 dpi and again downregulated at 21 dpi (Fig 4D). In the hippocampus *tgfβ1* expression was significantly down at 7 dpi. Unlike the SN, hippocampal *tgfβ1* levels returned to baseline levels by 10 dpi (Fig 5C).

### CA/09 infection upregulates M2 phenotype gene markers *arginase-1* and *csf1* in SN

Functionally, microglia/macrophages are known to exist in 2 states, deemed M1 or M2. The M1 phenotype is thought to exist when microglia are exposed to molecules that are considered to be proinflammatory such as IFNγ, TNFα, and IL-6. The M2 or “alternatively activated” phenotype occurs when microglia and are exposed to anti-inflammatory cytokines such as IL-4, IL-10, and IL-13 [26] and is characterized by the expression of arginase (*arg1*) [28]. Following H1N1 infection, we observed a delayed but significant increase in *arg1* gene expression that was first measured at 21dpi (Fig 4E), suggesting that the morphologically activated microglia observed in the brain of CA/09 infected mice are functionally expressing an anti-inflammatory or M2 phenotype. Another anti-inflammatory factor *csf1*, known to be increased to confer neuronal protection under inflammatory conditions and increase M2 phenotype among macrophages [29], was also found to be significantly increased at 7 and 10 dpi in the SN compared to saline controls (Fig 4F).

## Discussion

In this study, we demonstrated that the non-neurotropic CA/09 influenza virus, responsible for the 2009 influenza pandemic, is capable of inducing an encephalitic reaction in the brain. Our results demonstrated a significant increase in the numbers of microglia with activated morphology in both the *pars compacta* of the SN and the dentate gyrus of the hippocampus starting at 21 dpi; a phenomenon that persists through 90 dpi. In addition to the increase in activated microglial numbers, we also noted altered mRNA expression of a number of neurotrophic factors and cytokines/chemokine genes.

Microglia, which are derived from a macrophage lineage [30], act similarly to innate immune cells and play an active role in both CNS surveillance and support of neuronal function [11]. While actively surveying their microenvironment, microglia appear as small spheroid cells with long slender processes. However, upon exposure to noxious stimuli or other insults, they change their morphology into an “activated” state that is manifested by a dramatic increase in somal size and retraction and thickening of processes [31] and potentially the secretion of a number of pro- and anti-inflammatory factors [32,33]. Thus, functional plasticity of microglia is a property of its microenvironment and, depending on the molecules released, the functional state of microglia can change from an activated pro-inflammatory state (M1) to an alternative activated (M2) or acquired deactivated (M3) state [34,35]. Microglia in the M2 phenotypic state have been demonstrated to play an important role in repair and resolution of inflammation [32].

Our results demonstrated a significant increase in the numbers of microglia with activated morphology in both the *pars compacta* of the SN and the dentate gyrus of the hippocampus starting at 21 dpi; a phenomenon that persists through 90 dpi. Using qPCR analysis, we examined the mRNA expression of several genes implicated in both the activation and repression of an inflammatory response at 7, 10 and 21 dpi. These time points were chosen because they occur in the peak and recovery stages of the time course of CA/09 virus infection in mice, respectively (Fig 2). Of the genes examined, we found a significant down-regulation of the neurotrophin genes *bdnf* and *gdnf* at 7 dpi in the SN. The consequences of this reduction in neurotrophin expression are important, since these proteins both support neuronal health [36,37] and play an integral role in alleviating microglial activation [38]. However, this reduction in gene expression levels of neurotrophic factors was not accompanied by a loss of tyrosine hydroxylase (TH)-positive dopamine neurons in the SN. We also examined the expression of a number of proinflammatory genes including *il1β*, *il6*, *ptgs2* and *tnfα* at both 7 and 21 dpi and did not observe any significant alterations in gene expression, in the SN. The lack of proinflammatory gene expression in the brain led us to conclude that the morphologically activated microglia were not of an M1 phenotype characterized as secretory and inflammatory [39], but were likely serving a surveillance function, which is characteristic of the M2-type of microglia. [40]. This finding was further reinforced in the SN by significant increases in gene expression of *arg1*, *tgfβ1* and *csf1* genes that have been demonstrated to promote an anti-inflammatory environment in the CNS. Interestingly, these changes gene expression patterns were independent and non-similar to each other. Although *tgfβ1* expression is known to be upregulated following a number of CNS insults including ischemia, experimental allergic neuritis, or peripheral axotomy [41–43], we observed a decrease in expression at 7 and 21 dpi [44].

In many cases of inflammatory encephalitis there is a noted infiltration of peripheral lymphocytes into the CNS that have been hypothesized to occur due to a transient breach in the BBB [45]. Interestingly, the development of an encephalitic reaction following intranasal CA/09 infection occurred without breach of the BBB or T-cell infiltration into the brain parenchyma. Based on these results, we hypothesized that the increase in the number of microglia with activated morphology were, at least in part, secondarily attributable to the decrease in the intrinsically expressed neurotrophic factors [46] and the increased expression levels of circulating cytokines known to be involved with macrophage signaling following infections [47,48]. These could come from cytokine/chemokines released from T- and B-cells as well as endothelial cells in blood vessels lining the BBB [49–51]. This is important due to the possibility that the activated microglia may continue to serve a critical function after resolution of the peripheral inflammation caused by CA/09 infection. The hypothesis that T-and/or B-cells are critical mediators of this peripheral/central immune response could be tested using transgenic mice lacking T- and B-cells (RAG-1)[52,53] or through antibody-mediated depletion of specific immune cell populations [54,55].

Previously, we demonstrated that the highly pathogenic avian influenza virus A/VN/1203/04 was neurotropic and induced both an inflammatory pathology as well as induction of a number of parkinsonian pathologies including tremor, loss of tyrosine hydroxylase (TH) positive dopaminergic neurons in the SN and increased phosphorylation of alpha-synuclein [9,18]. We concluded that the intraparenchymal inflammatory response was secondary to the viral invasion. In this study, we demonstrated that non-mouse adapted CA/09 was non-neurotropic in mice, however, an encephalitic reaction in the CNS was observed, evident by the increased M2 phenotype microglial activity and changes in gene expression. Given that neuroinflammation contributes to the generation and progression of a number of neurodegenerative disorders [56], these findings have significant implications as they highlight the potential of many other non-neurotropic viruses to be probable initiators of pre-conditioning neuronal insults that may bestow neuroprotection by limiting inflammation for future CNS insults [57–59].

## Materials and Methods

### Ethics Statement

All of the experimental procedures in the animals were performed in accordance with the NIH Guide for the Care and Use of Laboratory Animals. These studies were carried out under protocol numbers 513 and 364 and were approved by the St Jude Children's Research Hospital IACUC under the auspices of Animal Assurance Number: A3077-01 on file with the Office of Laboratory Animal Welfare of the NIH.

### Animal Care and Use

Eight-week-old female C57BL/6J mice (Jackson Labs, Bar Harbor ME) were acclimated in our animal facility for a period of a week and maintained on a 12 h light/dark cycle with *ad libitum* access to food and water. All virus infections and animal treatments were performed in accordance with the NIH Guide for the Care and Use of Laboratory Animals and all protocols were approved by the IACUC of St. Jude Children’s Research Hospital, Memphis, TN.

### Preparation and inoculation of mice with CA/09 H1N1

A/California/04/2009 (CA/09) H1N1 virus was propagated in the allantoic cavity of 10-day-old specific pathogen-free embryonated chicken eggs. At 48 to 72 hours post-infection, allantoic fluid was harvested, clarified by centrifugation, and stored at −70°C. Tissue culture infectious dose 50% (TCID_50_) titers were determined using Madin-Darby canine kidney (MDCK) cells and evaluated by the method of Reed and Muench [60]. For infections, eight-week old mice were lightly anesthetized by isofluorane inhalation and intranasally inoculated with 10^3^ TCID_50_ of CA/09 in 25μl of phosphate-buffered saline (PBS). Following viral infection, mice were monitored for clinical signs of illness including physical appearance (hunched posture and disheveled coat appearance) and body weight was recorded every 48 hours after infection [61]. Mice that lost greater than 30% of their initial weight were humanely euthanized. On an average, the observed mortality rate was 15% over different infected cohorts.

### Influenza M gene expression

RT-PCR expression studies of the highly conserved influenza matrix (M) gene was used to determine viral presence in the brain tissues over the time course of CA/09 infection. The substantia nigra (SN), striatum, hippocampus and cortex regions of the mouse brain were microdissected at 7 and 21 days post-infection (dpi), processed to obtain mRNA, and used for the measurement of gene expression (n = 5). This method was used in conjunction with the immunohistochemistry methods to detect for viral neurotropism.

### Immunohistochemistry

CA/09 virus-infected C57BL/6J mice and appropriate-aged saline-treated controls were deeply anesthetized with an overdose of Avertin. Following the loss of the deep tendon and corneal reflexes, mice were transcardially perfused with physiological saline followed by 3% paraformaldehyde. The perfused brains were processed for paraffin embedding. Brains were sectioned on the microtome at 10μm thickness and mounted on polyionic slides (Superfrost-plus, Fisher Scientific). Deparaffinized sections were incubated with primary antibody for identification of dopaminergic neurons and microglia [mouse monoclonal anti-tyrosine hydroxylase (TH; Sigma-Aldrich; 1:500) and rabbit polyclonal Iba-1 (Wako Chemicals; 1:500)], respectively. The secondary antibodies included biotinylated mouse IgG (for TH, 1:1000) or biotinylated rabbit IgG (for Iba-1, 1:1000). Diaminobenzidine (DAB) or a VIP kit (Vector labs) reaction was used to yield a brown (TH) or a purple (Iba-1) color, respectively.

To detect viral protein, 3% neutral buffered paraformaldehyde perfused mouse brains and lungs were cryoprotected in 30% sucrose solution after which they were rapidly frozen in cryoprotective medium, sectioned at 20μm and thaw-mounted onto polyionic slides. Sections were stored at -20°C until immunostaining. Staining of these sections was conducted in accordance with the protocol suggested in the Mouse on Mouse (M.O.M.; Vector labs) kit. Viral protein was detected using an anti-NP antibody (Meridian Life Sciences; 1:500). Biotinylated mouse IgG (1:1000) was used as the secondary antibody and the NP was visualized using DAB.

### Quantification of Iba-1-positive microglia

Numbers of Iba-1-positive microglia in the SNpc were estimated using standard model-based stereological methods [62,63]. Counts of total and activated microglia [31] were estimated using Microbrightfield StereoInvestigator (MBF Biosciences, Williston, VT) and the optical fractionator method [64] using a Olympus BX-51 microscope and 100X objective. Stringent measures were adopted to classify Iba-1 positive microglia as resting or activated based on morphology [31,63]. Microglial cells were deemed as resting if they contained a small oval Iba-1-positive cell body that averaged 3 microns in diameter with long slender processes. Microglia were classified as activated when the cell body was slightly increased in size compared to resting microglia and had an irregular shape. Additionally, the processes on the activated microglia were shorter and had thickened processes.

### Measurement of blood-brain barrier integrity

Blood-brain barrier (BBB) integrity was measured by two different methods. In the first method, an intravenous injection of 2 mg FITC-albumin solution prepared by reconstituting 10mg of FITC-albumin (Sigma) in 1ml of sterile 0.9% saline solution and was injected into the tail vein of mice at a dose of 10ml/kg in saline administered controls or at 7, 10 and 14 dpi with CA/09. Two hours following the injection, the mice were deeply anesthetized with avertin, brains harvested and postfixed in 3% paraformaldehyde solution prior to sectioning on a cryostat at 20μm thickness. The mounted sections were coverslipped using Vectashield mounting medium for fluorescence and observed using fluorescence microscopy. Evidence for a leaky BBB was provided by using mice injected with LPS (2x 3 mg/kg) as a positive control (n = 3/group). Leaky BBB was observed by a general increase in fluorescence as well as the presence of extruded albumin from small blood vessels into the brain parenchyma [65]. Another method adopted to study the BBB integrity was by injecting sodium fluorescein dye intraperitoneally into mice at 2, 5, 7, 10 and 14 dpi (n = 4/group). Mice were injected intraperitoneally with 100μL of a sterile 10% (w/v) solution of sodium fluorescein (Sigma-Aldrich) in PBS. Ten minutes after injection, the mice were euthanized and cardiac blood was collected and perfused with 30mL of sterile PBS. Sodium fluorescein in the brain was quantitated by homogenizing brain tissue in 7.5% (w/v) trichloroacetic acid (TCA) followed by centrifugation and neutralization using 5N NaOH. Sodium fluorescein in serum was measured by mixing equal parts of serum and 15% (w/v) TCA, centrifugation and mixing the supernatant with a 1:1 solution of 7.5% TCA–5 N NaOH. Fluorescence in serum and brain samples was measured using a microplate reader and excitation at 485nm and emission at 530nm and concentrations were determined using standards ranging from 0.125μg/ml to 4 μg/ml. Values were normalized to fluorescein concentrations measured in serum and expressed as uptake ratios which was the ratio of the amount of sodium fluorescein measured in the brain to the amount measured in serum. The uptake ratio of sodium fluorescein was presented as a scatter plot [22,66].

#### Identification and Dissection of Brain Structures

Brain tissue from mice was dissected based on the following coordinates: SN and hippocampus (Bregma: -2.70 to -3.70).

### Real time quantitative RT-PCR

The substantia nigra (SN) and hippocampal regions of the mouse brain were microdissected at 7, 10 and 21 days post-infection (dpi) with CA/09 or saline administered controls (n = 5), homogenized and processed to obtain mRNA in accordance to the protocol outlined in RNAqueous-Micro kit (Ambion, Austin, TX). The isolated RNA extracted was converted to cDNA High Capacity RNA to cDNA kit (Applied Biosystems, Carlsbad, CA). This cDNA was subsequently used for qPCR analysis to quantify expression of different genes in the two brain regions. The gene expression Taqman assays were obtained from Life technologies. The ribosomal 18S (18S) and beta-actin genes were used as the normalizing/housekeeping gene for gene expression experiments. The alterations in gene expression have been expressed as 2^-ΔΔCt^ denoting fold-change in mRNA levels for each gene.

### Statistical Analysis

Statistical significance of data was determined using one-way analysis of variance (ANOVA) with Dunnett’s post-hoc test to compare different groups with controls; on GraphPad Prism (San Diego, CA). Data has been represented as mean ± standard error of the mean and a value of *p* ≤0.05 was determined as statistically significant.

## Supporting Information

S1 Fig Stereological Assessment of SNpc DA neuron number. The number of TH+ DA neurons in the SNpc was estimated using design-based stereology in saline administered C57BL/6J mice (Ctrl) and H1N1 infected mice at 21, 60 and 90 dpi. No change is TH+ SN neurons were observed at any timepoint (n = 8).
(TIF)

S2 Fig Appearance of microglia in the hippocampal dentate gyrus after intranasal administration of CA/09 H1N1. Representative images of sections through the rostral hippocampal dentate gyrus from saline-treated (A-C) or H1N1 [21dpi (D-F) & 60 dpi (G-I)-infected] demonstrate Iba-1 positive microglia with different morphology. The inset box demonstrates the magnified regions of the dentate gyrus (20X (B) and 40X (C)) to better demonstrate the morphology of the Iba-1 positive microglia. Microglia have a resting appearance characterized by a small nucleus and thin processes in saline-treated mice (A-C), while many microglial cells in mice treated with H1N1 have an “activated” morphology characterized by a larger cell body and shortened thickened processes. Scale Bars: A,D,G 200 μm, B,E,F 20 μm, C,F,I, 10 μm, J,K 10 μm.
(TIF)

S1 Table Relative Expression of inflammatory response genes in SN 7 and 21 dpi after CA/09 N1N1 infection. All results are compared to mice intranasally administered saline.
(DOCX)
