# Supplementary figures and images for: Induction of Microglia Activation after Infection with the Non-Neurotropic A/CA/04/2009 H1N1 Influenza Virus
Source: PLoS One. 2015 Apr 10;10(4):e0124047. doi: 10.1371/journal.pone.0124047 (PMC4393251; doi:10.1371/journal.pone.0124047)

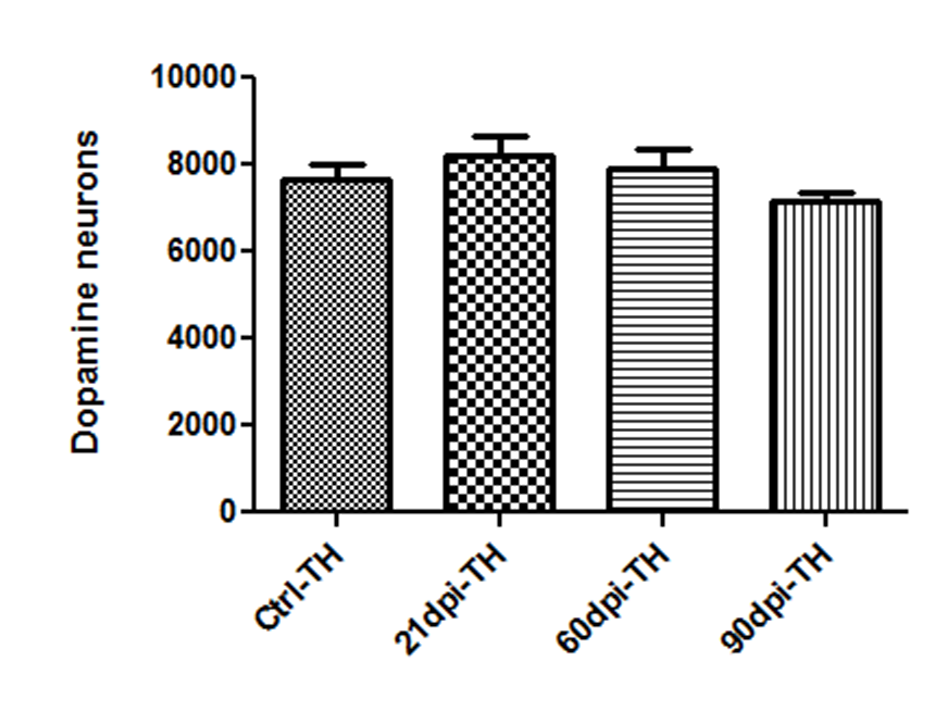

Supplement: S1 Fig — The number of TH+ DA neurons in the SNpc was estimated using design-based stereology in saline administered C57BL/6J mice (Ctrl) and H1N1 infected mice at 21, 60 and 90 dpi. No change is TH+ SN neurons were observed at any timepoint (n = 8). (TIF) [file pone.0124047.s001.tif]

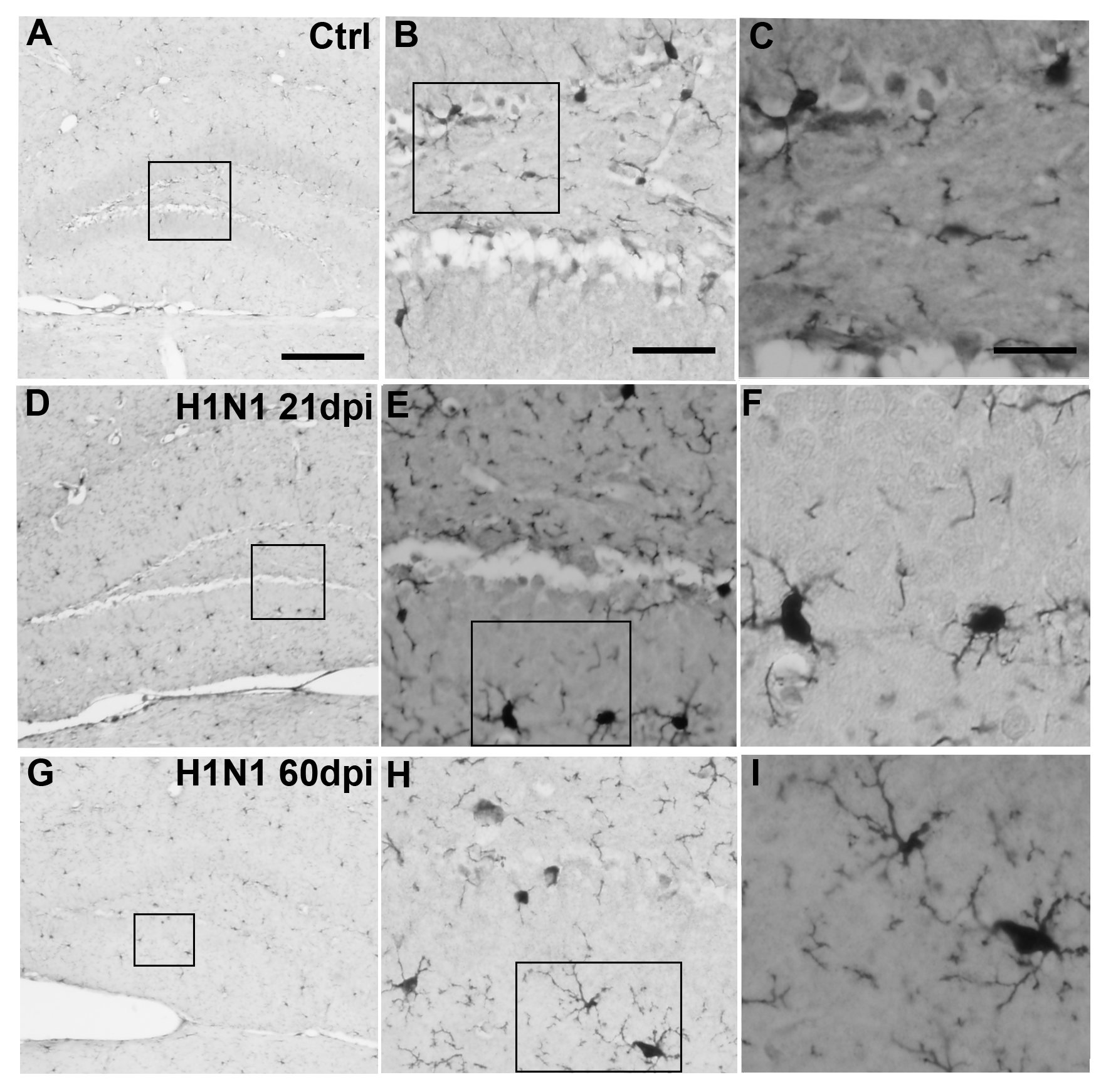

Supplement: S2 Fig — Representative images of sections through the rostral hippocampal dentate gyrus from saline-treated (A-C) or H1N1 [21dpi (D-F) & 60 dpi (G-I)-infected] demonstrate Iba-1 positive microglia with different morphology. The inset box demonstrates the magnified regions of the dentate gyrus (20X (B) and 40X (C)) to better demonstrate the morphology of the Iba-1 positive microglia. Microglia have a resting appearance characterized by a small nucleus and thin processes in saline-treated mice (A-C), while many microglial cells in mice treated with H1N1 have an “activated” morphology characterized by a larger cell body and shortened thickened processes. Scale Bars: A,D,G 200 μm, B,E,F 20 μm, C,F,I, 10 μm, J,K 10 μm. (TIF) [file pone.0124047.s002.tif]
