# Supplementary material for: Induction of Microglia Activation after Infection with the Non-Neurotropic A/CA/04/2009 H1N1 Influenza Virus
Source: PLoS One. 2015 Apr 10;10(4):e0124047. doi: 10.1371/journal.pone.0124047 (PMC4393251; doi:10.1371/journal.pone.0124047)
Supplement: S1 Table — All results are compared to mice intranasally administered saline. (DOCX) [file pone.0124047.s003.docx]

Suppl Table 1

Gene expression changes in the SN at 7 and 21 days post CA/09 H1N1 infection

| **Gene symbol** | **Control** | **H1N1 7dpi** | **H1N1 21dpi** |
| --- | --- | --- | --- |
| Ptgs2 | 1.01±0.1 | 1.26±0.22 | 1.34±0.11 |
| TNF-α | 1.01±0.11 | 0.75±0.23 | 1.3±0.19 |
| IL-6 | 1.03±0.09 | 1.02±0.04 | 0.92±0.03 |
| IL-1b | 1.01±0.12 | 1.30±0.36 | 1.26±0.18 |
| TLR2 | 1.04±0.2 | 1.01±0.07 | 0.97±0.13 |
| TLR5 | 1.00±0.04 | 1.05±0.17 | 0.88±0.13 |
| TLR6 | 1.01±0.08 | 1.00±0.05 | 0.64±0.07 |
| TLR9 | 1.02±0.15 | 1.05±0.16 | 0.70±0.1 |
| Tlr3 | 1.01±0.1 | 0.88±0.05 | 1.09±0.2 |
| Tlr4 | 1.09±0.3 | 0.85±0.18 | 0.94±0.13 |
| GFAP | 1.01±0.09 | 1.72±0.42 | 0.98±0.12 |
